# Morroniside Regulates Endothelial Cell Function via the EphrinB Signaling Pathway after Oxygen-Glucose Deprivation *In Vitro*

**DOI:** 10.1155/2022/6875053

**Published:** 2022-12-17

**Authors:** Tingting Liu, Songyang Zheng, Fangling Sun, Xin Tian, Zixin Zhu, Wenrong Zheng, Yufeng Wang, Jianguo Xing, Wen Wang

**Affiliations:** ^1^Department of Experimental Animal Laboratory, Xuanwu Hospital of Capital Medical University, Beijing 100053, China; ^2^College of Pharmacy, Xinjiang Medical University, Xinjiang, Urumqi 830054, China; ^3^Xinjiang Institute of Materia Medica, Xinjiang, Urumqi 830002, China; ^4^Beijing Institute for Brain Disorders, Beijing 100069, China

## Abstract

Proangiogenic treatment is a potential treatment for acute myocardial infarction (AMI). Morroniside was previously discovered to increase post-AMI angiogenesis in rats as well as the proliferation of rat coronary artery endothelial cells (RCAECs). However, the effects of morroniside on other endothelial cell (EC) functions and underlying mechanisms are unknown. To further clarify the vascular biological activity of morroniside, this work focused on investigating how morroniside influenced endothelial cell functions, such as cell viability, tube formation capacity, migration, and adhesion, and to explore the signaling pathway. Oxygen-glucose deprivation causes ischemic damage in RCAECs (OGD). In vitro investigations were carried out to explore the involvement of morroniside in EC function and pathways mediated by ephrinB. The results revealed that the number of BrdU^+^ cells and cell viability in the high-dose group were considerably greater than in the OGD group (*P* < 0.05). The ability of tube formation evaluated by total tube length, tube-like structural junction, and tube area was significantly higher in the morroniside group than in the OGD group (*P* < 0.001). Morroniside considerably improved migration and adhesion abilities compared to OGD group (*P* < 0.05, *P* < 0.01, *P* < 0.001). The protein expression levels of the ephrinB reverse signaling pathway were substantially greater in the morroniside group than in the OGD group (*P* < 0.05, *P* < 0.01). In conclusion, the current study demonstrated that morroniside modulates endothelial cell function via ephrinB reverse signaling pathways and provided a novel insight and therapeutic strategy into vascular biology.

## 1. Introduction

Cardiovascular disease (CVD) has been identified as the major factor resulting in mortality worldwide [1]. Ischemic cardiomyopathy, as a frequently-occurring myopathy of the heart, is primarily caused by inadequate supplies of nutrients and oxygen, resulting in an infarcted myocardium. Despite notable breakthroughs in revascularization attempts, such as percutaneous catheter intervention and surgical revascularization. Therapeutic angiogenesis has gained extensive academic attention [2]. Improving neovascularization is a therapeutic strategy for rescuing tissues from critical ischemia. Angiogenesis, the process through which endothelial cells (EC) sprout in the existing vasculature, followed by migration, proliferation, and tube creation, is critical in microvascular development and revascularization following myocardial ischemia (MI) [3]. Understanding angiogenesis is critical for developing novel therapeutics for MI injuries.

There are several routes involved in the pathological process of angiogenesis. Increasing evidence has shown that ephrinB reverse signaling participates in the regulation of the angiogenic process. Upon activation, the intracellular domains of ephrinB ligands can be phosphorylated via Src family kinases (SFKs), allowing them to bind to adaptor proteins such as Nck2 and activate downstream signaling molecules [4]. Many of the signaling pathways initiated by ephrinB signaling in endothelial cells have been identified and linked to various endothelial cell functions [5].

Morroniside is one of the most abundant iridoid glycosides extracted from *Cornus officinalis*. Morroniside has previously been shown *in vivo* to promote endothelial progenitor cell proliferation, increase vessel density, and improve cardiac function after acute myocardial infarction (AMI). Besides, morroniside was also corroborated to stimulate the rat coronary artery endothelial cells (RCAECs) proliferation *in vitro* [6]. However, little is known about the morroniside's impact on other endothelial cell activities and the underlying mechanisms. In this work, we conducted a further study on cell viability, capillary tube formation, endothelial cell migration, adhesion, and related signaling pathways. These findings provide an experimental foundation for better understanding the cardiac angiogenesis-regulatory mechanisms of morroniside.

## 2. Materials and Methods

### 2.1. Drug Preparation

This work purified morroniside, a sarcocarp extract from *C. officinalis* (Tong-Ren-Tang, Beijing, China), according to a prior procedure [7]. HPLC (high-performance liquid chromatography)-based measurement revealed a 98.5% final purity.

### 2.2. Culture of Rat Coronary Artery Endothelial Cells

RCAECs (Cat No: CP-R081) were purchased from the ProCell Life Science&Technology Corporation (Wuhan, China). RCAECs were cultured in high-glucose Dulbecco's modified Eagle's medium (DMEM) (Gibco, Thermo Fisher Scientific Inc., Waltham, MA, USA) that contained penicillin-streptomycin (1%; Gibco, ThermoFisher Scientific), 10 ng/ml endothelial growth factor (EGF, PeproTech, Rocky Hill, USA), as well as 10% fetal bovine serum (FBS) (Biological Industries, Kibbutz Beit-Haemek, Israel) under a humidified (5% CO_2_ and 95% air) condition at 37°C.

### 2.3. Oxygen Glucose Deprivation (OGD)

For the *in vitro* ischemia simulation, cells were subjected to hypoxic exposure (95% N_2_ and 5% CO_2_) and subsequently an 8 h incubation in glucose-free DMEM (Gibco, Thermo Fisher Scientific). Control cells, on the other hand, were incubated in normoxic (5% CO_2_ and 95% air) context for 8 h. A group of the cells was accomplished under five groups. Control cells were subjected to normal culture, while OGD cells were subjected to 8 h OGD treatment. For the three OGD + morroniside groups, the cells were pretreated with 1, 10, or 100 *μ*M morroniside for 24 h and then subjected to OGD for 8 h.

### 2.4. Fluorescence Assay

In immunofluorescence staining, the RCAECs were seeded into 48-well plates at a density of 20,000 cells/well. For 5-bromo-2-deoxyuridine (BrdU) assay, this work immobilized cells following an 8 h incubation using BrdU (10 *μ*M, Sigma-Aldrich, St. Louis, MO, USA). Then, the present work adopted immunocytochemical approach for quantifying the BrdU-positive cells. As a first step, RCAECs were immobilized for 20 min in paraformaldehyde (4%), and then treated for 17 min using 2 N HCl at 37°C. After thrice PBS washing and a 1 h blockage at an ambient temperature using the donkey serum (5%, v/v, Jackson ImmunoResearch, Philadelphia, USA), an overnight incubation proceeded using the primary anti-BrdU mouse antibody (1 : 200; Roche, Indianapolis, USA) within 5% donkey serum involving PBS under 4°C. Cells were later incubated for a 2 h period using ALexa Fluor 594-labelled secondary antibody (1 : 400, Life Technologies, Carlsbad, USA) at an ambient temperature. Afterwards, the cellular nuclei were labelled using mounting medium with 4,6-diami-dino-2-phenylindole (DAPI) (Abcam, Cambridge, USA). To label F-actin, cells were fixed with 4% formaldehyde, followed by permeabilization using 0.1% Triton X-100. Fluorescein 555-labeled phalloidin (Abcam) was added to stain F-actin. Following the cellular nucleus labelling using mounting medium with DAPI (Abcam), a Ni-U fluorescence microscope (Nikon) was utilized for the cellular visualization.

### 2.5. Cell Viability Analysis

This experiment adopted cell counting kit-8 (CCK-8, Dojindo, Shang Hai, China) for assessing survival in line with specific instructions. In brief, RCAECs at 4 × 10^4^/well density were seeded into 24-well plate. CCK-8 solution (50 *μ*l) blended with a medium (500 *μ*l) was added to the cells, followed by 2 h incubation under 37°C. The absorbance (OD) value was detected with the microplate reader (Infinite M200, TECAN) at 450 nm.

### 2.6. Tube Formation Assay

150 *μ*l of Matrigel basement membrane matrix (growth factor enriched) (Becton, Dickinson and Company, Franklin Lakes, NJ, USA) was coated into each well of the 48-well plates in a sterile environment, and no air bubble was introduced, followed by 60-min incubation under ambient temperature for allowing the transformation of Matrigel into a gel. Subsequently, Matrigel was added with RCAECs (2 × 10^4^/well). At last, the plates were added with 300 *μ*l serum-free medium for further incubation under 37°C and 5% CO_2_ conditions. Each experiment was carried out twice to take the average for separate experiments. At 8 h postincubation, four fields were randomly selected from each well to take the images, then Image-Pro Plus software was utilized to determine tube length, branch points, and tube area.

### 2.7. Scratch Assay

Scratch assay was conducted to assess RCAECs migration. Briefly, this work grew RCAECs (1 × 10^5^/well) within the 6-well plates to approximately 90% density, and each well was scratched with a sterile 200 *μ*l pipette tip to make a straight wound approximately 1 mm wide in the middle of the cells after synchronizing the cells for 24 h. For determining cell migration, images were captured using an Olympus X71 fluorescence microscope at the indicated time. The area of scratch was determined with ImageJ software (National Institutes of Health, Bethesda, MD, USA).

### 2.8. Cell Migration

The 8 *μ*m 24-well Transwell chambers (Corning, Lowell, MA, USA) were employed for evaluating cell migration. In brief, 5 × 10^4^ cells were resuspended in serum-free medium before being put on top of each chamber insert. 10% FBS-containing DMEM was added into the bottom chamber to be the chemoattractant. Thereafter, one cotton swab was utilized to remove cells there were still on the upper membrane. After 24 h, methanol was used to fix the cells migrating via pores, followed by crystal violet staining.

### 2.9. Cell Adhesion

3 *μ*g/ml fibronectin was added onto the 96-well plates for overnight incubation under 4°C, followed by 2 h blocking using 1% BSA under 37°C. Later, cells (5 × 10^4^/well) were added into the 96-well plate involving serum-free buffer of the reagent under test (100 *μ*l/well), and then subjected to a 30 min incubation under 37°C. Following mild plate rinsing using PBS at 100 *μ*l per well, 100% ice-cold methanol (100 *μ*l/well) was utilized to immobilize the adherent cells at 4°C for 5 min. After one wash with distilled water, the cells were stained for 15 min with crystal violet (50 *μ*l/well; 0.5% (w/v) in 20% (v/v) methanol) at room temperature. Subsequently, the wells were washed several times with water and left to dry overnight on a bench. Crystal violet was extracted for 10 min at room temperature by adding 50 *μ*l/well of 10% (v/v) acetic acid, and the absorbance was read at 562 nm. An equally-treated plate was utilized for determining background, albeit without cells. Cell adhesion in the absence of a reagent was considered 100% [8]. For visualization, DAPI (Sigma-Aldrich) was added for cell counterstaining, followed by observation with an Olympus X71 fluorescence microscope.

### 2.10. Western Blot Analysis

RCAECs (1 × 10^5^/well) were added into the 6-well plates, which were employed for western blotting. A radioimmunoprecipitation assay (RIPA) (Applygen, Beijing, China) involving protease inhibitors (complete mini, Roche) was utilized for extracting the proteins. The BCA assay (Applygen) was employed for estimating the levels of proteins. After protein isolation on SDS-PAGE, they were shifted to nitrocellulose membranes (Millipore). Membranes were blocked with 5% nonfat dry milk for 2 h at room temperature and incubated overnight at 4°C in 5% milk/tris-buffered saline containing 0.1% Tween-20 (TBST) with the following primary antibodies: rabbit anti-p-ephrinB (1 : 1000, Cell Signaling Technology, Beverly, MA, USA), rabbit anti-Nck-2 (1 : 1000, Abcam), rabbit anti-p-FAK (1 : 500, Abcam), rabbit anti-VE-cadherin (1 : 1000, Abcam), rabbit anti-Integrin*α* 5 (1 : 1000, Abcam), and rabbit anti-GAPDH (1 : 2000, Cell Signaling Technology). The membranes were incubated with appropriate peroxidase-conjugated secondary antibodies at 1 : 2000 for 2 h at room temperature, followed by the visualization of immunoreactive proteins on the film using an ECL kit (Millipore, Temecula, CA, USA).

### 2.11. Statistical Analysis

Data are expressed as mean ± standard deviation (SD) and were statistically analyzed using SPSS 20.0. A one-way ANOVA followed by Tukey's test was used for comparisons between multiple groups. *P*  <  0.05 was considered statistically significant.

## 3. Results

### 3.1. Morroniside Regulates Proliferation, Viability, and Tube Formation of RCAECs

**Figure 1:**
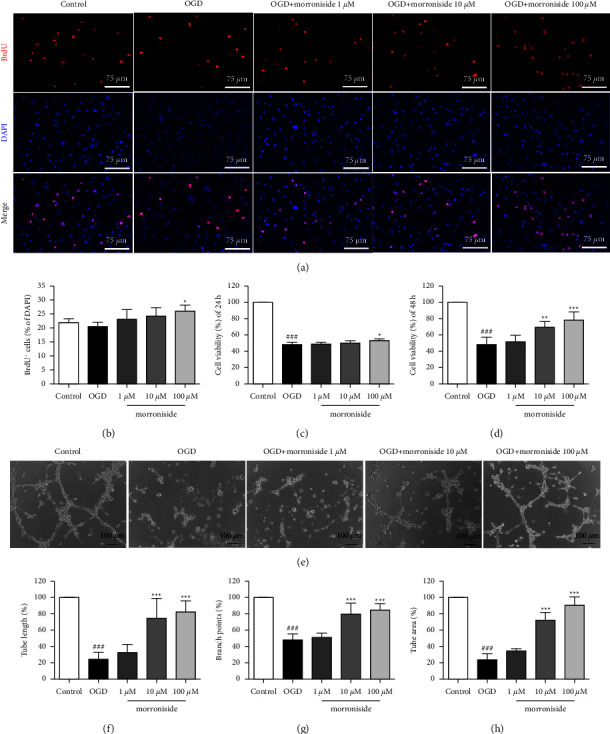
Morroniside promotes proliferation, cell viability, and tube formation of RCAECs. Typical IHC images showing BrdU ^+^ cells *in vitro* (a). BrdU^+^ cell quantification (*n* = 5) (b). Cellular viability analysis of RCAECs using a CCK-8 assay after the treatment with morroniside 24 h and 48 h (*n* = 6) (c, d). Typical tube formation images obtained at 8 h after cell seeding onto Matrigel (e). The image J was utilized to quantify branch points, total tube length, and tube area (*n* = 5) (f–h). Results were represented by means ± SD.  ^###^*P* < 0.001 in comparison with the control.  ^*∗*^*P* < 0.05,  ^*∗∗*^*P* < 0.01, and  ^*∗∗∗*^*P* < 0.001 relative to the OGD group.

We previously demonstrated that morroniside increased the number of Ki67^+^ RCAECs *in vitro* [6]. Ki67 immunohistochemistry, on the other hand, can only yield information on the proliferative state of the cells because Ki67 is expressed in all phases of the cell cycle except for G0 [9]. To further estimate the rate of the cell proliferation, we labelled S-phase with BrdU. The 100 *μ*M morroniside group exhibited pronouncedly elevated BrdU^+^ cell quantity in contrast to the OGD group (Figures 1(a) and 1(b)). The viability of RCAECs following morroniside therapy was then evaluated. As indicated in Figures 1(c) and 1(d), morroniside dramatically promoted cell viability compared to the OGD group. Tube formation experiments are a key method for evaluating the ability to undergo angiogenesis *in vitro*. To further determine the proangiogenic effects of morroniside, this work measured total tube length, tube-like structural junction count, and tube area to evaluate the tube formation. As illustration in Figures 1(e)–1(h), compared to the OGD group, the tube formation significantly increased following morroniside at concentrations of 10 *μ*M and 100 *μ*M treatment, respectively. These results provide direct evidence that morroniside promoted proliferation, cell viability, and tube formation in RCAECs.

### 3.2. Morroniside Regulates Migration of RCAECs

**Figure 2:**
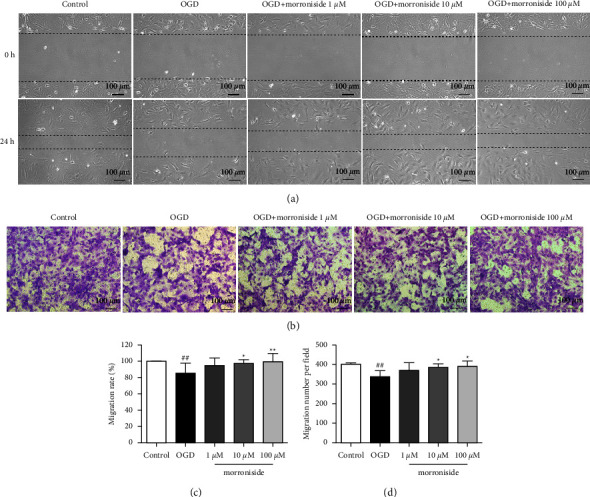
Morroniside promotes migration of RCAECs. Representative images of cells wound healing from each group (a). Representative images of Transwell insert from each group (b). Quantification of the migration rate in wound healing assay (*n* = 6) (c). Quantification of the migrating cell count per field in the Transwell assay (*n* = 6) (d). Results were represented by means ± SD.  ^##^*P* < 0.01 relative to the control.  ^*∗*^*P* < 0.05 and  ^*∗∗*^*P* < 0.01 relative to the OGD group.

The migration of RCAECs treated with morroniside *in vitro* was determined using a wound healing model. Morroniside significantly increased RCAEC migration (Figures 2(a) and 2(c)). We also performed a Transwell migration assay to study the migratory ability of RCAECs (Figures 2(b) and 2(d)). These results were consistent with the migration assay conducted using the wound healing model. Compared with the OGD group, Transwell migration was prominently enhanced in the 10 *μ*M and 100 *μ*M morroniside groups. These results indicated that morroniside promoted the migration of RCAECs.

### 3.3. Morroniside Regulates Adhesion of RCAECs

**Figure 3:**
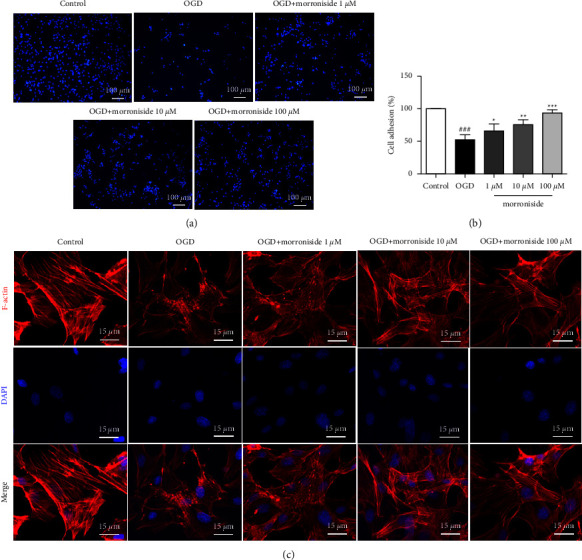
Morroniside promotes adhesion of RCAECs. Representative images of adherent cells on FN-coated wells (a). The quantification of adherent cell number by 100% of the control group (*n* = 6) (b). Representative images of F-actin structures stained by fluorescently labeled phalloidin as well as nuclei stained by DAPI (c). The arrow stands for F-actin bundles, whereas arrowhead stands for F-actin clumps. Results were represented by means ± SD.  ^###^*P* < 0.001 relative to the control;  ^*∗*^*P* < 0.05,  ^*∗∗*^*P* < 0.01, and  ^*∗∗∗*^*P* < 0.001 relative to the OGD group.

Angiogenesis is a multistep process that includes cell-matrix adhesion, cell migration, and tube formation. As tube formation and cell migration depend on cell-extracellular matrix adhesiveness, we evaluated the effects of morroniside on endothelial cell adhesion to fibronectin, a major substrate for endothelial cells. Results show that treatment with morroniside increased the fibronectin-adherent RCAECs in a dose-dependent manner (Figures 3(a) and 3(b)). Cell adhesion is the interaction of cells with the actin cytoskeleton that connects them to the extracellular matrix. To investigate whether morroniside induced F-actin alignment, fluorescently-labeled phalloidin was utilized to visualize F-actin cytoskeleton. As shown in Figure 3(c), the control group had the majority of the F-actin bundles. Oxygen-glucose deprivation model resulted in the disruption of the F-actin network, resulting in actin clumps within the cytoplasm. Cells treated with morroniside showed fewer F-actin clumps and more F-actin bundles than the OGD group. As a result, morroniside enhanced RCAECs adhesion and stabilized F-actin filaments.

### 3.4. Morroniside Regulates ephrinB Reverse Signaling Pathway-Related Protein Levels

**Figure 4:**
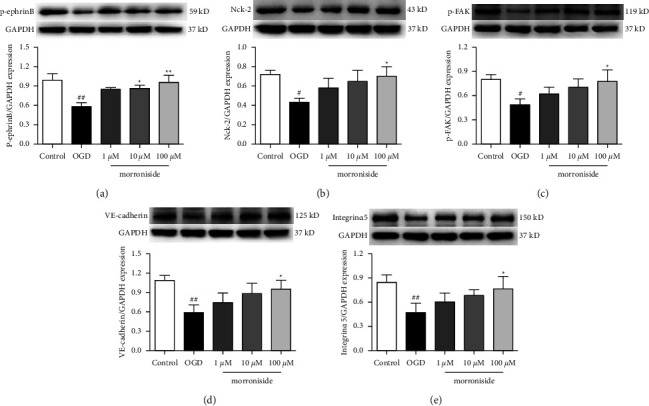
Morroniside promotes the expression of ephrinB reverse pathway-related proteins. Typical WB images and p-ephrinB expression quantified relative to GAPDH (*n* = 4) (a). Typical WB images and Nck-2 expression quantified relative to GAPDH (*n* = 4) (b). Typical WB images and p-FAK expression quantified relative to GAPDH (*n* = 4) (c). Typical WB images and VE-cadherin expression quantified relative to GAPDH (*n* = 4) (d). Typical WB images and integrin*α*5 expression quantified relative to GAPDH (*n* = 4) (e). Results were represented by means ± SD.  ^#^*P* < 0.05 and  ^##^*P* < 0.01 in comparison with the control group.  ^*∗*^*P* < 0.05 and  ^*∗∗*^*P* < 0.01 in comparison with the OGD group.

Compelling evidence indicates that the ephrinB reverse signaling pathway drives endothelial cellular processes, such as proliferation, adhesion, and migration after initiation [5, 10]. In this work, the p-ephrinB level decreased remarkably following OGD compared to the control group. The p-ephrinB level was elevated pronouncedly by treating with morroniside (Figure 4(a)). The phosphorylation of ephrinB ligands enables the binding of the adaptor protein Nck2 and activates the downstream proteins of focal adhesion kinase (FAK), VE-cadherin, and integrin*α*5 [4]. Next, the Nck2, p-FAK, VE-cadherin, and integrin *α*5 protein expressions were assessed. Resembling the expression outcome for p-ephrinB, the control group had decreased expressions of Nck2, p-FAK, VE-cadherin, and integrin*α*5 compared with the sham group. Morroniside administration significantly enhanced the levels of these proteins (Figures 4(b)–4(e)). As implied by the foregoing findings, the ephrinB reverse signaling probably serves as a mediator for morroniside's role in the endothelial cell function.

## 4. Discussion

Re-establishment of the blood supply to the myocardium after AMI is partly dependent on angiogenesis. Neovascularization is a critical element in the ischemic myocardium repair [11, 12]. In our prior work, morroniside has been corroborated to promote endothelial progenitor cell proliferation, increase vessel density, and improve cardiac function following AMI in rats [6]. The focus of this work were morroniside's effects on the endothelial cell function *in vitro* and its potential mechanisms. Our findings suggest that morroniside appears to facilitate the functions of proliferation, cell viability, tube formation, migration, and adhesion of RCAECs. The ephrinB reverse signaling pathway may be involved in the processes underlying these effects.

ECs are responsible for forming capillary tubes, which is their distinguishing properties and also the essential condition to establish the blood flow-routing continuous vascular lumen. Tube formation can be the rapid and quantitative approach for determining *in vitro* angiogenesis ability [13]. Our results demonstrated that a capillary-like tubular structure grew more thickly after treatment with morroniside. Furthermore, the morroniside treatment also increased the amount of BrdU ^+^ cells and cell viability following OGD. These results support our previous findings that morroniside enhances *in-vivo* angiogenesis [6]. Based on the general concept, ECs migration is an essential component of angiogenesis [14]. We explored how morroniside impacted the migration and discovered that the migration ability of RACECs was diminished after OGD injury and that the treatment with morroniside facilitates cell migration. Aside from the tube formation and migration, another crucial function of ECs is adhesion property. The dynamic integrin-mediated adhesion of ECs to the surrounding cell-extracellular matrix is critical for angiogenesis in the pathological condition. A wealth of evidence indicates that angiogenic migration persistently depends on the endothelial cell adherence to the cell-extracellular matrix [15]. Ruan and colleagues demonstrated that the enhanced focal adhesion contributes to the angiogenesis after OGD injury [16]. Following that, we evaluate the effect of morroniside on RCAECs adhesion to fibronectin and found that RACECs treated with morroniside were more adhesive than those in the OGD group. Due to the concurrent research, fibronectin participates in the cell attachment to the substrate localized adjacent to actin filament bundles and their termini [17]. Communication between the actin filaments and focal adhesions is crucial for cell adhesion and migration. Our findings revealed that F-actin was malaligned following OGD, while treatment with morroniside stabilized F-actin filaments. Our *in vitro* study, for the first time, clearly demonstrated morroniside's role in the RCAEC viability, tube formation, adhesion, and migration.

Reverse signaling of ephrinB has an important impact on regulating EC function. Inducing ephrinB reverse signaling by EphB receptor promotes angiogenesis [10]. In the current study, a marked p-ephrinB elevation was obvious after the treatment with morroniside. Park et al. revealed that ephrinB enhanced integrin-induced cells adhesion; moreover, integrins represented the transmembrane receptors activating signaling while causing cell invasion [18]. Furthermore, activating integrins accelerate focal adhesion to connect with the actin cytoskeleton, which is required for driving cell migration [19]. The ephrinB-Nck2 complex can activate focal adhesion kinase (FAK) and has an important impact on the integrin signal [20]. Depending on our findings shown in Figure 4, the p-FAK, Nck2, and integrin *α*5 levels were prominently raised following the morroniside therapy. It is well known that VE-cadherin represents the critical adhesive molecule in endothelial adhesive junctions, and there are several processes that influence the VE-cadherin-targeting endothelial junction stability [21]. The morroniside group exhibited evidently elevated VE-cadherin levels compared with the OGD group, which supported our research on the adhesion ability and F-actin filaments. As implied by these findings, the ephrinB reverse signaling is a potential mediator for the endothelial cell function-regulatory mechanism of morroniside. Combining our prior study of the regulation of morroniside on the VEGF/VEGF receptor 2 signaling pathway to promote cell proliferation at the cellular level by culturing RCAECs [6], we hypothesize that ephrinB signaling pathway may be a novel important target of morroniside. Hence, further research is needed to fully understand the underlying mechanisms of morroniside's action and to clarify its biologically active components more in depth.

## 5. Conclusions

Collectively, we demonstrated that morroniside promoted endothelial cell functions of proliferation, tube formation, migration, and adhesion. Morroniside-induced increases in ephrinB reverse signaling components are thought to have mediated the function regulation mechanism. Based on our previous research, this study further clarifies the vascular biological activity of morroniside through regulating the endothelial cell function and revealed the prospective targets.

## Data Availability

The data utilized for supporting our results are included in this work.
